# A Tribute to a Visionary Scientist—John R. David—Richard Pearson Strong, Professor of Tropical Public Health, Emeritus, Harvard

**DOI:** 10.3390/pathogens13030208

**Published:** 2024-02-27

**Authors:** Jeffrey J. Shaw, Abhay Satoskar

**Affiliations:** 1 Department of Parasitology, Institute for Biomedical Sciences, University of Sao Paulo, Av. Prof. Lineu Prestes, 1374, Sao Paulo 05508-000, SP, Brazil; 2 Department of Pathology, Ohio State University, Columbus, OH 43210, USA; satoskar.2@osu.edu

There are rare individuals whose insatiable curiosity and boundless intellect propel them into multiple frontiers of science, leaving an indelible mark on the fields that they venture into. This Special Issue is a modest testament to one such luminary. John David is a scientist whose contributions span the spectrum of discovery, from unravelling the world of cytokines to uncovering the sources, novel treatments, and control of leishmaniases. After listening to a talk by the late Philip Marsden at the Harvard School of Public Health on Leishmaniasis, it was suggested to him that mucocutaneous could be related to delayed hypersensitivity reactions. At Philip’s invitation, John travelled to Bahia to observe cases for himself, and this cemented his interest in the disease. Thus, cytokines and allergy led him to leishmaniasis!

This Special Issue is more than a compilation of scientific papers; it is a mosaic of tributes, with each paper written by esteemed colleagues, mentees, and friends who were fortunate enough to know or be influenced by the brilliance of such a scientist. Each paper is a unique brushstroke and contributes to the portrait of John, whose impact resonates across disciplines. We would like you to reflect on the profound legacy of this extraordinary person as you read the papers included in this Special Issue, covering the mosaic of leishmaniasis research. 

The contributions delve into the front lines of ground-breaking research on these devastating and neglected diseases that mutilate and kill. They demonstrate the enormous breadth of research that is involved in leishmaniasis, reflecting John David’s approach to science: all doors are open! There are contributions on epidemiology, chemotherapy, clinical immunology, and molecular biology that illuminate the mysteries that surround this devastating disease. A special legacy John’s ground-breaking discovery that cytokines are the key to understanding the multitude of pathologies associated with the many parasite species that cause this disease and may lead to novel treatments. This Special Issue hopes to inspire those undertaking research in leishmaniasis and promote others to apply their specialist knowledge to a spectrum of neglected diseases known as leishmaniases. In their many forms, these diseases cause enormous suffering, and are amongst the most difficult to control and cure. However, above all, this Special Issue is a reflection of a mentor, a collaborator, and a friend, whose influence extends far beyond the laboratory

